# Various Applications of ZnO Thin Films Obtained by Chemical Routes in the Last Decade

**DOI:** 10.3390/molecules28124674

**Published:** 2023-06-09

**Authors:** Mariuca Gartner, Hermine Stroescu, Daiana Mitrea, Madalina Nicolescu

**Affiliations:** Institute of Physical Chemistry “Ilie Murgulescu”, Romanian Academy, 202 Splaiul Independentei, 060021 Bucharest, Romania

**Keywords:** ZnO thin films, sol-gel, seed layer, doping, nanostructures, composite materials

## Abstract

This review addresses the importance of Zn for obtaining multifunctional materials with interesting properties by following certain preparation strategies: choosing the appropriate synthesis route, doping and co-doping of ZnO films to achieve conductive oxide materials with p- or n-type conductivity, and finally adding polymers in the oxide systems for piezoelectricity enhancement. We mainly followed the results of studies of the last ten years through chemical routes, especially by sol-gel and hydrothermal synthesis. Zinc is an essential element that has a special importance for developing multifunctional materials with various applications. ZnO can be used for the deposition of thin films or for obtaining mixed layers by combining ZnO with other oxides (ZnO-SnO_2_, ZnO-CuO). Also, composite films can be achieved by mixing ZnO with polymers. It can be doped with metals (Li, Na, Mg, Al) or non-metals (B, N, P). Zn is easily incorporated in a matrix and therefore it can be used as a dopant for other oxidic materials, such as: ITO, CuO, BiFeO_3,_ and NiO. ZnO can be very useful as a seed layer, for good adherence of the main layer to the substrate, generating nucleation sites for nanowires growth. Thanks to its interesting properties, ZnO is a material with multiple applications in various fields: sensing technology, piezoelectric devices, transparent conductive oxides, solar cells, and photoluminescence applications. Its versatility is the main message of this review.

## 1. Introduction

Zinc oxide is a versatile material with a wide range of applications in different fields such as chemistry, materials science, biology and nanotechnology due to its simple and environmentally friendly synthesis, biocompatibility, and high chemical stability [1]. Over time, as a result of their impressive properties, involving a wide band gap of 3.37 eV, exceptional electron mobility (1 to 200 cm^2^/Vs), and an exciton binding energy of 60 meV [2,3], ZnO has been comprehensively explored in different forms for certain applications [4]. Thus, depending on the synthesis methods, ZnO can be obtained as: bulk [5,6], nanostructures [7,8], thin films, or hybrid materials [9,10]. The properties of ZnO-based materials can be tailored and enhanced by controlling and optimizing several parameters (solution concentration [11], dopant level [12,13], synthesis [14,15,16,17], and annealing temperature [18] or pH [19]). In the last decade, the development of nanostructures with various morphologies [20,21,22,23,24,25,26,27] such as: nanowires, nanorods (NR), nanoflowers, nanosheets, nanobelts, nanoneedles, nanoplates, has gained tremendous attention, being used in biological applications [8,28,29,30,31,32,33,34]: bioimaging, biosensing, antibacterial and drug delivery agents. In particular, ZnO thin films have optical (photoluminescence) [33,35,36,37,38,39,40,41,42], electrical (thermoelectric [43,44,45,46], piezoelectric [47,48,49,50,51,52,53]) and biological (antimicrobial [54,55,56], antibacterial [57,58,59]) characteristics, which make them excellent candidates for the development of optoelectronic [60,61,62,63,64,65], piezoelectric [66,67,68], transparent conductive oxides (TCO) devices [69,70,71], ultraviolet (UV) photodetectors [13,72,73], solar cells [74,75,76,77,78], photocatalysts [79,80], gas sensors [81,82,83,84,85,86,87,88,89,90] or biosensors [91,92,93]. The previous papers have discussed ZnO films with varying morphologies (e.g., nano-particles or nanorods) that were grown on a wide range of substrates, using different chemical methods for different applications. In 2022 alone, at least nine reviews were published on ZnO, suggesting the topicality of a review about materials with Zn in various combinations and forms.

**Figure 1:**
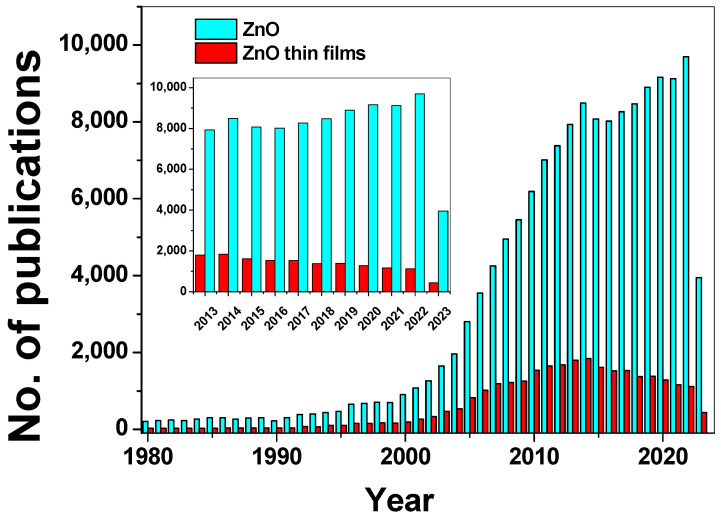
Evolution of the number of scientific papers related to the search of “ZnO” and “ZnO thin films” phrases published between 1980–2023. Inset: illustration of the last decade. Source: Scopus (accessed on 12 May 2023).

To illustrate the continuous progress and growing research interest in ZnO, a graph depicting the annual number of publications on ZnO and ZnO thin films from 1980 to 2023 (Figure 1) was included, obtained with SCOPUS. This graph highlights a significant growth in the publication numbers between the years 2000 and 2015. The inset in Figure 1 shows a constant interest in this topic, for the last decade.

The novel viewpoint of this review is that it starts from ZnO nanoparticles (NPs), continuing with mono- and mixed ZnO layers, covering doped and codoped ZnO, and finally, considering Zn as a dopant by itself.

## 2. Preparation Methods of ZnO Films

Over the years, various physical and chemical methods have been used to prepare ZnO films. In this chapter, we focus on the chemical methods and highlight some unique aspects based on recent literature findings. Some of the best-known chemical methods for ZnO film preparation include sol-gel (SG) (using dip-coating and spin-coating for deposition), hydrothermal (HT), chemical bath deposition (CBD), and successive ionic layer adsorption and reaction (SILAR).

### 2.1. Sol-Gel Synthesis

**Figure 2:**
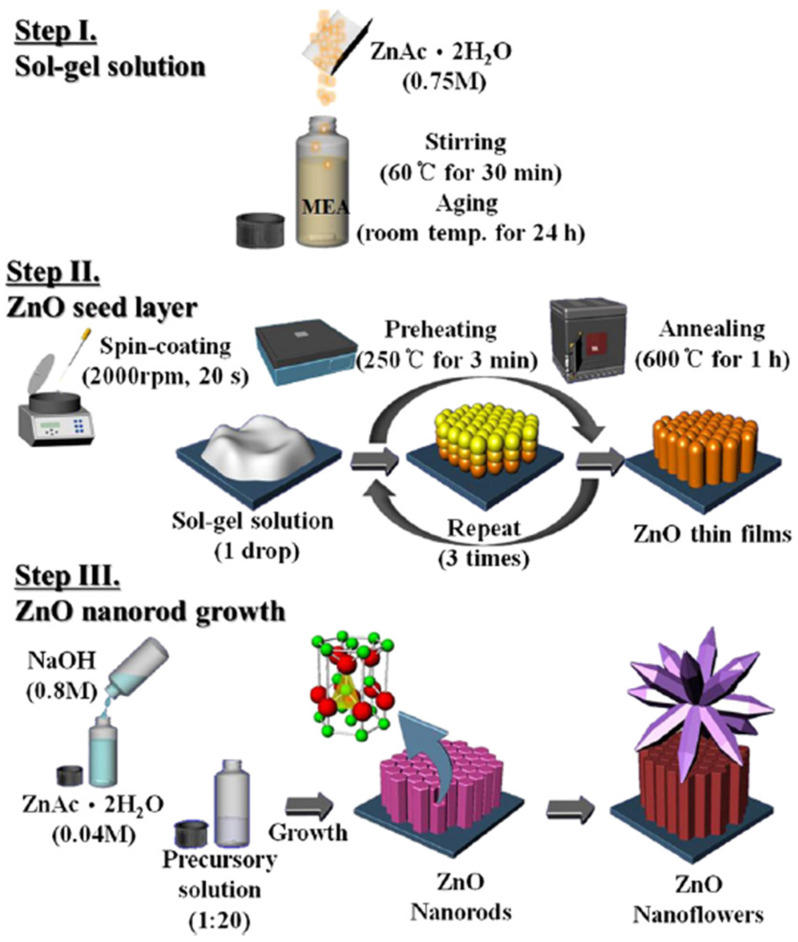
Graphic illustration of sol-gel solution preparation, ZnO seed layer deposition and ZnO nanorod growth. Reprinted from [94] with permission from Elsevier.

The SG technique is one of the most popular deposition methods, extensively used in the last years to prepare inexpensive ZnO thin films. A graphic illustration (Figure 2) of the SG solution preparation, the spin-coating deposition of a seed-layer, and the HT growth of ZnO nanorods is presented in Ref. [94].

The synthesis conditions of the SG method can modify the film surface and at the same time, they can improve the catalytic and chemical sensing properties of the film. The variation of precursor concentrations is among the best-known procedures to obtain the desired properties.

Other factors that may influence the final properties of the films are the microwave treatment of the sol [95] or the polymer modifiers [96,97,98,99]. The application of microwave heating in the SG method is a good way to reduce the preparation time and to obtain nanostructured films at lower temperatures [96]. On the other hand, hydroxypropyl cellulose (HPC) or ethylcellulose (EC) added into the sol-gel precursor solution led to high photocatalytic activity, high chemical sensitivity [96] and the control of the particle size [97] and film porosity [98].

Shankar [99] showed that adding the monomer of the polymer poly(vinyl alcohol) (PVA) along with dehydrated zinc acetate led to an enhanced carrier concentration in ZnO nanorods and in turn increased the sensitivity to ethanol detection at room temperature.

Jang [100] proved that the modification of ZnO with siloxane polymers increased the sensitivity of ethanol detection at room temperature.

Significantly enhanced charge transport characteristics necessary in sensor applications were obtained using conjugated polymer (CP) semiconductors [101], contributing to the next generation of high-performance organic field-effect transistor (OFET) sensors.

### 2.2. Hydrothermal Method

The hydrothermal method is usually performed to grow ZnO NRs in an autoclave, from a water-based solution at specific conditions of high pressure and temperature. A previously deposited SL on the substrate induces the formation and the growth of NRs with a specific morphology [94,102,103,104].

The growth characteristics of nanorods and nanowires are as many as their use in specific applications [105,106,107,108], with requirements regarding the density, length, and thickness of the nanowires. These requirements can be met by varying the temperature (higher temperatures generally lead to faster growth rates), the pressure (the use of a low-pressure can result in the growth of long and thin nanowires), the growth time (affecting the length of the resulting nanorods or nanowires), the number of deposition cycles, etc.

### 2.3. Chemical Bath Deposition

**Figure 3:**
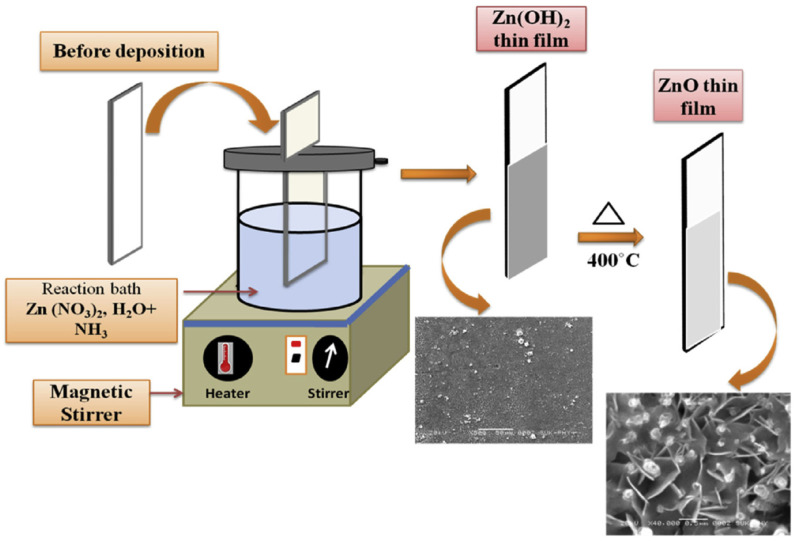
Schematic diagram of CBD technique used to obtain ZnO thin films. Reprinted from [113] with permission from Elsevier.

Chemical Bath Deposition is a process used to deposit thin films onto a substrate which involves the use of a chemical solution that contains precursors for the desired material [109,110,111,112]. The substrate is immersed in the solution and a reaction occurs at the surface of the substrate, resulting in the deposition of a thin film. Figure 3 [113] illustrates the step-by-step process of ZnO thin film formation using the CBD technique.

The CBD process offers several advantages, including low cost, ease of use, and the ability to deposit films on a wide range of substrates, including plastics and glass. The process can also be used to deposit films with controllable thickness and composition, making it useful for a variety of applications, including solar cells, sensors, and optoelectronics.

### 2.4. Successive Ionic Layer Adsorption and Reaction

**Figure 4:**
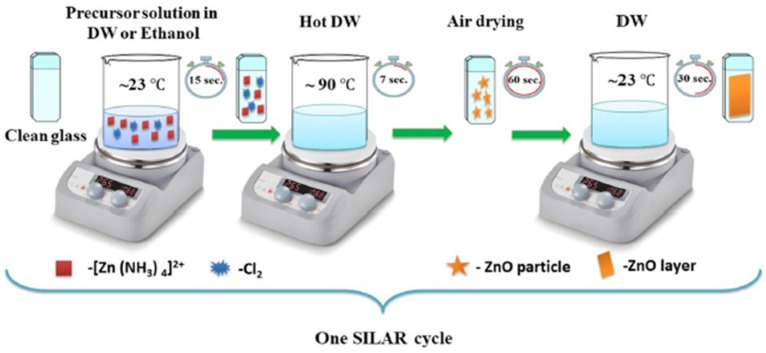
Graphical representation of SILAR method used to obtain ZnO thin films. Reprinted with permission from [116]. Copyright 2022, Yergaliuly et al.

The successive ionic layer adsorption and reaction deposition method was first described by Nicolau [114] and Ristov et al. [115]. It is an ion-by-ion deposition at room temperature, which is conducted by alternately immersing the substrate, first in the cation solution and then in the anion solution of a given compound, each step being followed by the rinsing of the excess solution with deionized water. A schematic diagram of this process is presented in Figure 4 [116]. The thickness and the morphology are better controlled than in other related methods, such as CBD [117]. The thickness is monitored using the number of successive immersions, as well as the concentration and the type of the reactant precursors, tuning the thickness from the nm to μm range, depending on their intended use.

Among other precursors, zinc chloride is proven to be the most suitable precursor for Al-doped ZnO thin films [118], with good morphological, optical, and electrical properties, for applications in optoelectronic devices.

The number of SILAR cycles plays a major role in obtaining a texture suitable for photoelectrochemical applications [119], with a preferential orientation along the (002) plane. With the increase of the deposition cycles from 60 to 120, a more compact growth of the nanorods was observed, which can be explained by the formation of more nucleation centers on the substrate. A second increase from 120 to 180 cycles leads to an agglomerated growth with a deviation from vertical shape, with a negative impact on the photoelectrochemical performance.

### 2.5. Other Chemical Methods: Spray Pyrolysis, Inkjet Printing, Chemical Vapor Deposition (CVD)

Another chemical method is spray pyrolysis [120,121,122], popular for its low-cost results, in which the precursor solution is deposited on a heated substrate using a high-velocity gas carrier. The film thickness and properties can be controlled by adjusting the concentration of the precursor solution, the spray rate, and the substrate temperature.

Spray pyrolysis was used to obtain ZnO thin films. The observed increase of the bandgap energy is a result of the ZnO SL presence, which also induces changes in the morphology from flower-like (~6 μm diameter) for samples without SL to horizontal nanorods (~0.8 μm length) in the presence of the SL [123]. This method was also used to obtain undoped and Mg-doped ZnO films on indium tin oxide (ITO) SL [124]. The choice of a different material for the SL had, as a result, the selective nucleation and further growth of ZnO grains which exhibited a good H_2_ sensing response, even more, improved by doping ZnO with Mg.

This method is a relatively simple and cost-effective process used to deposit a wide range of materials, such as oxides, nitrides, and metals, for various applications, including sensors, photovoltaics, and catalysis.

A variation of the spray pyrolysis method is the Inkjet printing, where using jets with different pressures, the material is deposited on previously designed shapes [125,126,127,128]. The inkjet printing technique offers several advantages for ZnO thin film deposition. It allows for precise control of the thickness and morphology of the film, as well as the ability to pattern the film with high spatial resolution. It is also a low-cost method that can be used to obtain ZnO thin films on a variety of substrates, including flexible ones. Inkjet printing is a promising technique for the fabrication of ZnO thin films for different applications, such as solar cells, sensors, and electronic devices.

The CVD method is used to grow different films on a substrate using a chemical reaction between vapor phase reactants [129,130]. The CVD method allows for the deposition of high-purity films with exceptional uniformity over large areas and enables the growth of complex structures (nanowires, thin films, multilayers, etc.), with precise control over composition, thickness, and orientation [131,132,133].

In summary, there are several effective methods for preparing ZnO films, including SG, CVD, spray pyrolysis, etc., each with its advantages and disadvantages depending on the desired film properties and application requirements.

## 3. ZnO as Seed Layer (SL)

In this chapter, a brief overview of the importance of SL is emphasized, together with some applications in which the SL is essential in tuning the final material properties.

An SL is typically defined in the literature as a very thin layer deposited on different substrates to further assist the growth of specific nanostructures, such as nanorods, nanowires, nanosheets, etc. The ZnO SL quality is critical, because it plays a significant role in inducing a preferential orientation of the ZnO films grown on its surface, and at the same time it influences their morphology, diameter, and crystallinity.

The deposition of a SL is also important in obtaining a good adherence of a subsequent layer to the substrate. In various applications, such as piezoelectric materials, for example, the nucleation sites of a SL offer the possibility to grow nanowires of different dimensions and directions, depending on the SL thickness.

**Figure 5:**
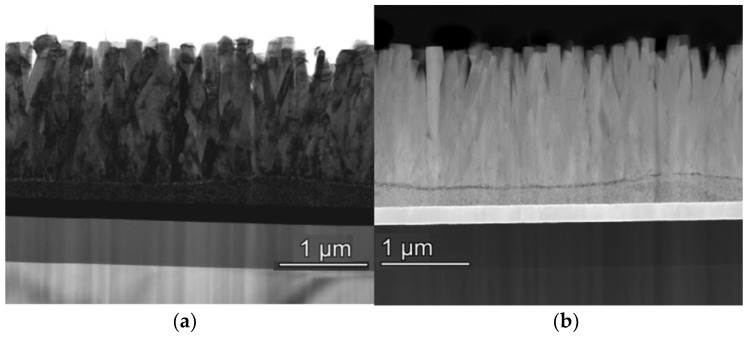
BF TEM (**a**) and HAADF images from ZnO NWs (**b**) on Au substrate. Reprinted from [47] with permission from Elsevier.

Especially in the hydrothermal method (HT), a SL is necessary before growing 2D structures. This technique was successfully used to obtain columnar ZnO grains of 1300–1700 nm thickness and 80–160 nm in diameter, grown from a ZnO SL deposited by the SG method [47]. Figure 5 shows (a) the bright-field (BF) transmission electron microscopy image and (b) the high-angle annular dark-field (HAADF) image of ZnO NWs film grown on Au/Ti/SiO_2_/Si substrate by HT method [47].

Good quality ZnO SL is also used to reduce the lattice mismatch between a substrate and the layers to be grown on it, developing vertically aligned ZnO nanorods with a good density and crystallinity control [134,135].

Many works have investigated the effect of deposition parameters on the initial SL in obtaining vertically oriented nanorods. The SL quality is highly influenced by the preheating temperature and annealing conditions. The preheating stage is the thermal treatment performed after each deposition on a multi-layer SG film and its temperature varies depending on the different precursor solutions used in the deposition process.

As shown in [136], a preheating temperature of 400 °C is necessary to decompose the organics by-products and to obtain vertically aligned ZnO nanorods, while in [137] it is stated that the preheating process is providing the necessary energy for the nucleation and growth kinetics of the ZnO film, directly affecting the crystalline quality of the final film. The influence of different preheating temperatures on the growth of vertical nanorods is studied in [135,138] and in this case, the best nanorods alignment along the c-axis was found in the samples with a SL obtained at 350 °C preheating temperature.

In other words, the effect of ZnO SL annealing temperature on the growth of ZnO nanorods has been studied. A higher temperature (500 °C) led to an increase in the diameter and length of ZnO NR, due to the larger size of the SL grains [139]. UV sensors were produced based on SLs obtained at different annealing temperatures (100 ÷ 500 °C) [140] with the highest response for the sample annealed at 500 °C.

An increase from one to five successively deposited ZnO layers results in a thicker and denser SL on the substrate, when using the SG spin coating technique [141]. In the case of the CBD method, the increase of the number of adsorbed atoms provides a larger growth area for ZnO nanowires, and the highest density and rod-like structure of ZnO nanowires is obtained for the sample with five depositions of ZnO SL [141]. When ZnO nanowires were grown on the thinner SL, they had a flower-like structure [141].

Even though most thermal treatments are performed to improve the final properties of the samples, the annealing of the ZnO SL was reported in some cases to have a negative impact on the device performances, in applications such as NO sensors [142]. The annealing process enabled a larger diameter of the nanorods and lower porosity and, consequently a few percent decrease in gas sensing response in comparison with the as-deposited ZnO nanorods.

Banari et al. [108] have studied the UV photodetection properties of ZnO nanorods grown by two-step (spin coating and hydrothermal) method on SLs, with thickness varying in the range of 50–125 nm. Depending on the successive number of spin-coating depositions (3, 5, 7, and 9), increasing the SL thickness first leads to an increase in the carrier concentration from three to five layers, followed by a decrease in the last two cases. The high value of the carrier concentration was assigned to the surface defects and oxygen vacancies in the ZnO films.

In summary, the SL is a critical factor as it affects the nucleation, diameter, length, and uniformity of the resulting nanostructures.

## 4. ZnO in Composite Thin Films

**Figure 6:**
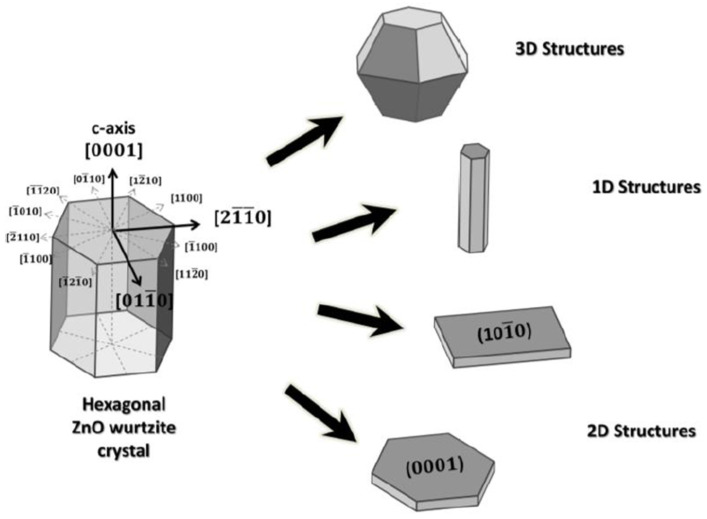
The growth directions of ZnO wurtzite crystal and possible morphologies. Reprinted from [143]. Copyright 2017, Leonardi, S.

The possible growth directions and the morphologies of ZnO (1D, 2D and 3D) are illustrated in Figure 6 [143]. In this chapter, we focus on different double-layer composites, with respect to the overall improvement of the composite thin films’ properties.

There are different combinations of double layers that contain ZnO and are efficient in the development of many applications. Such examples are presented in Table 1 for sensor applications.

**Figure 7:**
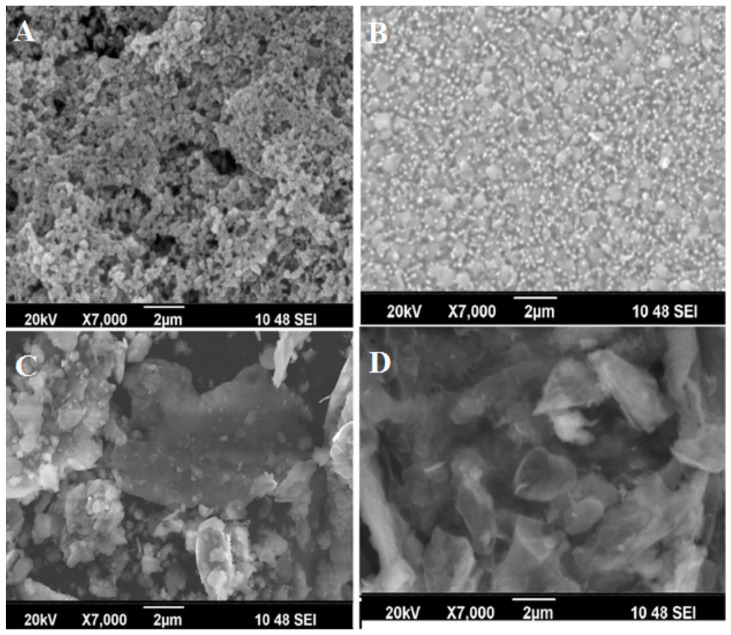
SEM images of (**A**) AZO, (**B**) AZO:rGO (2 wt.%), (**C**) AZO:rGO (4 wt.%), and (**D**) AZO:rGO (6 wt.%) composite thin films. Reprinted from [160] with permission from Elsevier.

***ZnO-graphene*** is an interesting and promising combination. Graphene and its derivatives present nonlinear optical properties (NLO), with high absorption and dispersion properties, with the ultrafast optical response, which makes them suitable for the application of the mode-locked lasers [157]. ZnO has strong second and third-order nonlinear optical features [158,159]. Sreeja et al. [160] showed that adding reduced graphene oxide (rGO) to the AZO layers leads to an increase in the absorption and decrease of the optical band gap, an effect that intensifies with the increase of the rGO concentration. The increase in the percentage of rGO from 2 to 6 wt.% leads to an enhanced formation of sheet-like structures in rGO, which subsequently merge with agglomerated ZnO particles, as could be seen in Figure 7 [160].

***ZnO-graphene*** is also used in gas sensors [161,162] because it improves their selectivity, shortens the recovery time, and operates at lower temperatures [162]. At the same time, ***this combination*** managed to mitigate electromagnetic radiation (with around 30 dBs) in the domain of 10–20 GHz [163], the attenuation being a function of the ZnO/graphene nanoplatelets ratio and of the frequency employed.

***ZnO-polymer*** can form very efficient piezoelectric coatings on flexible metallic substrates. Chelu et al. [47] showed that a vertical ZnO nanowires (ZnO NWs) array grown on flexible Ti substrate by the hydrothermal method at low temperature and covered with a layer of poly(methyl methacrylate) (PMMA) leads to high values, above 120 pC/N, of the piezoelectric coefficient *d_33_*. With such values, it is possible to foresee that PMMA/(ZnO NWs)/Ti nanostructures open the way towards integration in wireless or defense technologies and in wearable or implantable biomedical systems as efficient harvesters.

Gen-Wen Hsieh et al. [164] demonstrated that the composite dielectric film of poly(dimethylsiloxane)-PDMS-elastomeric silicone and zinc oxide tetrapod display remarkable sensing performance in the capacitance change and a pressure sensitivity of 2.55 kPa^−1^ over that of pristine polymer sensors, enabling a minimum detectable pressure of only 1.0 Pa. At the same time the composite has a rapid response and reliable sensing stability for over 1000 cycles. By introducing stress-sensitive additives of zinc oxide nanostructures, PDMS-ZnO composites may provide the basis for potential applications in touch sensing, electronic skin and sensitive wearable healthcare devices.

Another example of the role of ***ZnO-polymer*** composites is their use in medicine in sutures, dermal fillers, or stents applications [165]. Venkatesh et al. [166] used polylactic acid (PLA) and polypropylene (PP) polymers in urinary stent applications. Their combination with ZnO increased the antibacterial properties and polymer degradation.

The combination of **ZnO-Poly**(butylene adipate-co-terephthalate) (PBAT) is very useful in mechanical, thermal, and biological activity for food packaging [167] showing superior antimicrobial activity against *Escherichia coli* and *Staphylococcus aureus*. The tensile strength in the nanocomposite film with 10 wt.% ZnO enhanced to 45.0 MPa compared to 37.9 MPa of pure PBAT film, as well as increased thermal stability due to the good dispersion of ZnO nanoparticles in the PBAT matrix.

Another application was recently presented by B.C. Kang [162], in which ***ZnO NW, polymers, and carbon*** were used together in Wearable Pressure/Touch Sensors Based on Hybrid Dielectric Composites of Zinc Oxide Nanowires (NWs)/Poly(dimethylsiloxane) (PDMS) and Flexible Electrodes of Immobilized Carbon Nanotube (CNT) Random Networks. The incorporation of ZnO NW into PDMS increased the sensitivity of the composite in low-pressure regions, from 1.32 × 10^−4^ Pa^−1^ to 8.77 × 10^−4^ Pa^−1^. This effect appears due to the enhancement of piezoelectricity induced by ZnO NW on flexible CNT electrodes.

Combining the high charge carrier mobility of ZnO with the good film-forming properties of the polymer, the ZnO-polyethyleneimine (PEI) composite layer served as a cathode buffer layer for organic and perovskite solar cells [161]. Power conversion efficiency of the composite is improved compared to that of each individual ingredient and the performance of the perovskite as a solar cell increases from 10.05% to 11.76%. Chen et al. [168] also used PEI for doping ZnO for an efficient electron transport layer (ETL) in solar cell applications. At 7 wt.% PEI, they obtained vertical transport and the power conversion efficiency improved to 4.6% from a value of 3.7% of the corresponding device with pristine ZnO.

Another category of examples is the class of multi-component films based on ZnO, such as p-CuS-ZnS/n-ZnO heterostructures, prepared by SG [169] which can produce non-toxic, stable UV photodiode with an excellent rectifying behavior and very fast response.

## 5. Zn as Dopant

Zn can be interesting not only as a basic element in the ZnO films but also as a dopant introduced in other oxidic films. In Table 2 the role of Zn as a dopant is exemplified for SG films, as pointed out by recent works.

The examples above can be expanded with films prepared by other methods than SG, such as: *Spray pyrolysis* [179,180,181,182,183]; *Chemical bath deposition* [184,185,186]; *thermally vacuum evaporation* [187], and others [188,189,190]. When oxide layers are doped with Zn, their concentration plays an important role in improving parameters in various applications. The optoelectronic devices and solar cells have a better performance due to the enhancement of refractive index with the Zn concentration increase (1–5%) [175] and with the increase of smoothness of the surface morphology as the Zn wt.% grows to 10 wt.% [180].

In some cases, ZnO may have a positive effect only when it is in a small amount, while when its concentration is slightly higher, the effect is negative. Such a case is presented by Sheikh et al. [191] for Polyether block amide (PEBA) nanocomposites doped with ZnO. The effect of low ZnO (≤0.5%) concentration on the thermal and mechanical properties of prepared PEBA/ZnO nanocomposite thin films is very good but with increasing the concentration to 1%, it weakens due to agglomeration of the nanoparticles.

## 6. Doped and Codoped ZnO Films; p-Type Conductivity

Through the SG method, it is relatively easy to prepare stable n-type ZnO, but it is very difficult to obtain a p-type material due to the generation of donor-type defects which compensate for the charge of acceptor dopants (self-compensation effect) and the low solubility of the acceptor dopant ions [192]. According to Li et al. [193], p-type ZnO is characterized by a low concentration and mobility of holes, making it unstable over time. This instability leads to the tendency of the p-type ZnO to revert to n-type at room temperature within a specific time interval.

Thus, undoped ZnO shows n-type conductivity due to intrinsic defects such as interstitial Zn and oxygen vacancies. By doping and co-doping, p-type ZnO can be obtained through three fundamental approaches: (a) replacing Zn with the group I and IB elements (Li, Na, K, Ag, Cu), (b) by doping with group V elements (N, P, As, Sb) which replace oxygen in the lattice or (c) co-doping with donors and acceptors (Li-Ni, In-N, Al-N, F-Ag) [193,194,195]. In recent years, group I elements have been reported to possess better dopant behavior than group V or group III elements in terms of acceptor, and donor level, respectively [196,197].

**Figure 8:**
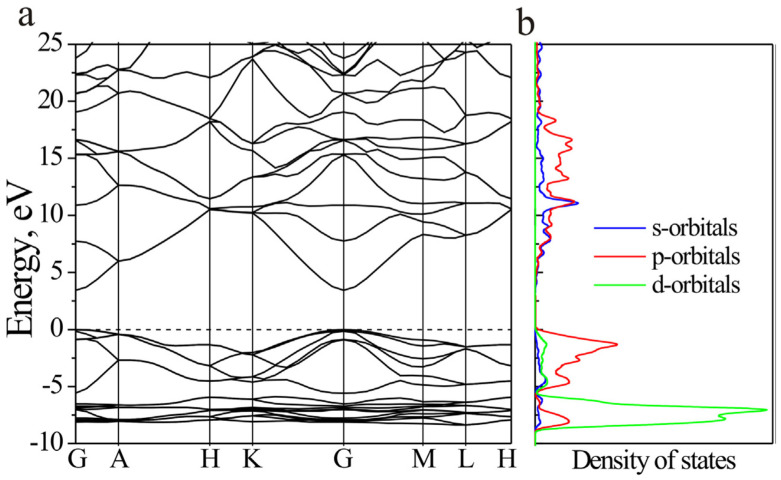
Band structure (**a**) and partial density of states (**b**) of ZnO. Reprinted from [198] with permission from Elsevier.

An extensive discussion on the ZnO band structure, the partial density of states (PDOS), and the lattice parameters are presented in [198] and illustrated in Figure 8a,b.

Li is considered the most suitable element from group I (Li, Na, K) to produce p-type ZnO modifying the strain effects and energy levels through the replacement of Zn with Li [199,200]. The reason for this is the small ionic radius of Li of 0.68 Å, which is very close to the ionic radius of Zn (0.74 Å). As a result, Li can occupy the Zn vacancy (V_Zn_) and induce the desired effects [201,202,203].

According to [204,205], another option is to dope ZnO with Na ions (0.95 Å), which replace Zn ions (Na_Zn_) and create a shallow acceptor state [205].

Also, it has been reported that apart from the ionic radius that can affect doping, the substrate material also plays a role in the doping of ZnO. For example, p-type behavior was thus obtained for Li-doped ZnO deposited on a silicon substrate, and n-type behavior was obtained for ZnO deposited on a glass substrate [199,203].

Maksimov [206] has reported that obtaining p-type ZnO materials via anion substitution, by replacing oxygen with other group VI elements (S, Se, Te) is used for the fabrication of photovoltaic devices [206,207].

ZnO has been doped with transition metals (such as Mn, Ni) to obtain dilute magnetic semiconductors (DMS) for applications in spintronic devices [199] and also has been doped with Ni or F elements to fabricate high-quality humidity sensors [208,209].

Co-doping of ZnO is reported to be feasible due to the strong attractive acceptor-donor interaction, which overcomes the repulsive interactions between the acceptors and leads to the formation of acceptor-donor-acceptor complexes [197].

In summary, based on the literature and the structural, morphological, elemental, optical, and electrical analysis, the p-type conductivity of ZnO (doped/co-doped) is attributed to the formation of an impurity band above the maximum of the valance band, resulting in a reduction of the band gap and a decrease in the energy of ionization of the acceptor (Li, Na, P, N) [194,199,200,201,205] respectively of the donor (Al, In, Ni) [196,199,207] highlighted in the spectroscopic analysis through the red shift in the UV emission [197,202].

The Raman spectra showed an increase in the intensity of phonon mode E1(LO) [197,200] which is associated with impurities and the formation of defects such as oxygen vacancies, demonstrating that the doping occurred.

A widening of the band-gap is usually observed for ZnO doped with donors, while a reduction of the band gap was noticed by doping with acceptors [197,199,205,207,210].

The change of the lattice parameters indicates that the dopant (in the form of ions) replaces the Zn ions and was incorporated into the ZnO lattice (confirmed by XRD, XRF, XPS measurements) [192,194,199,204,206,211].

The Hall, Seebeck or the current–voltage (I–V) measurements indicated that, usually, the undoped ZnO film exhibited n-type conductivity, while the doped ZnO films generally exhibited p-type conductivity with low carrier concentration [192,193,194,196,199,201,205,209,210,211,212,213].

Repeated measurements after a period of time (4–12 months) demonstrated that the conduction type is stable over time [199,211,212]. The p-type conductivity in thin films is generated by free O^2−^ vacancies and interstitial Zn atoms or substitution sites of Zn^2+^ ions.

In addition, the SG method [192,199,211,214] has proved to be an attractive technique for obtaining p-type ZnO films among others due to the low deposition costs compared to other more expensive methods such as RF magnetron sputtering [210,212] pulsed laser deposition [215] or spray pyrolysis [196,213].

The ionic character of elements used for doping ZnO is presented below in Table 3.

## 7. Applications

In the present section, a special attention is given to the applications of ZnO thin films prepared by sol-gel and hydrothermal methods.

**Figure 9:**
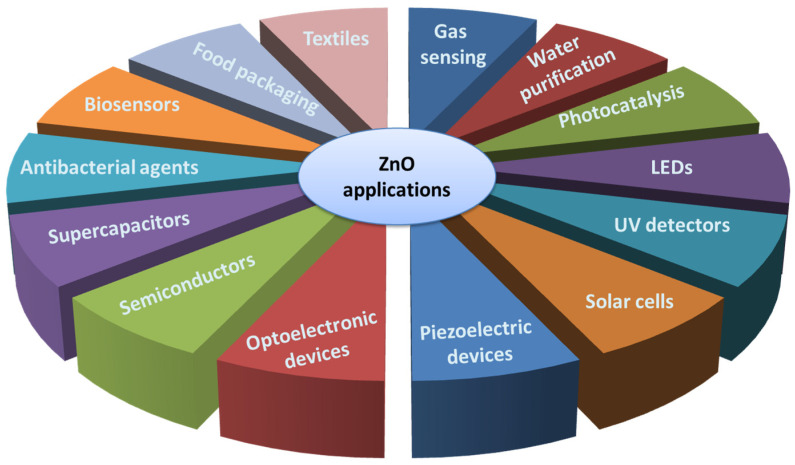
Applications of ZnO in different fields.

The versatility of the material is demonstrated in Figure 9.

An overview of the SG-ZnO applications in the literature of the last ten years is presented in Table 4.

In the previous sections, we mentioned the applications every time we considered the different types of layers and their preparation methods. This is obvious in Table 1, which is dedicated to sensor applications of composite layers containing Zn. Nevertheless, additional remarks are in order, regarding fluorescence because it is one of the most widely used detection mechanisms in many fields such as biology, biophysics, biochemistry, genomics, proteomics, drug discovery, disease diagnostics, and environmental analysis. A few recent examples are presented below:

### 7.1. Medical Field

In the reference [231], the status of 1D ZnO in vitro as bio-detection supports is summarized, as well as the challenges and future outlook concerning their application as enhanced *biomedical detection* platforms are presented.

**Figure 10:**
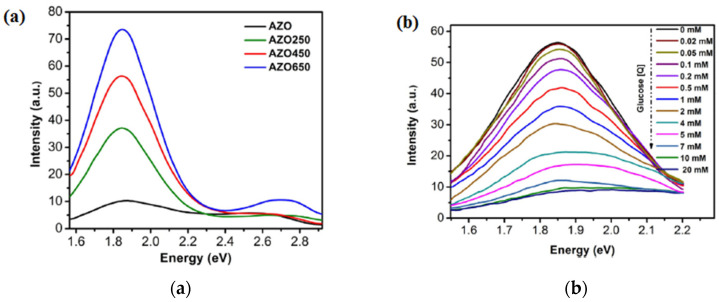
(**a**) PL spectra for as-grown and annealed AZO samples, (**b**) AZO450 PL spectra before and after glucose exposure at different concentrations in the presence of GOx. Reprinted from [30] with permission from Elsevier.

Al-doped ZnO (AZO) thin films, annealed at different temperatures (250 °C, 450 °C, and 650 °C for 1 h in air) are used for the detection of glucose based on *fluorescence quenching* [30]. From the AZO450 PL spectra (Figure 10) in the presence of glucose at different concentrations and immobilized with glucose oxidase (GOx), a systematic decrease in the PL intensity is observed with the increase in concentration from 20 μM to 20 mM.

In 2014, ZnO nanoparticles (NPs) have been employed for fluorescence lifetime imaging in human skin [233] and in the same year, Wolska et al. [234] have shown that rare earth (RE) elements can activate ZnO NPs to work as biomarkers, for medical visualization. ZnO NPs possess biocompatibility with the living organism and if they are doped with RE element, their route inside the organism can be monitored by the luminescence of RE atoms.

### 7.2. Antibacterial Field

The *antibacterial activity* of SG ZnO films presented by Kaviyarasu et al. [59] with different concentrations of ZnO particles (100–600 μg/mL) was successfully used against Gram-positive and Gram-negative bacteria (*S. pneumonia*, *S. aureus*, *E. coli* and *E. hermannii*). At the same time, the photocatalytic activity of ZnO under sunlight increases the degradation rate of Rhodamine-B (RhB), which is one of the common water pollutants emitted by textile and paper industries.

This *biological application* became more attractive due to their *duality in toxicity: benefits in drug delivery and the antibacterial effects*, as underlined in [20].

## 8. Summary, Conclusions, and Future Prospects

This review covers the major recent results on materials based on Zn, highlighting low-cost preparation methods (like chemical ones, especially SG). It focuses on the versatility of Zn in different combinations: as thin films (doped, undoped) for SL or main layer, as a thin film in a multilayer stack, as a component in a mixed thin film, or as a dopant in other films. A material with improved properties can be achieved by controlling and tailoring its morphology, crystallinity, and porosity.

The importance of the SL and its properties for obtaining thin films have been discussed, its presence being imperative for the growth of nanostructures with different orientations. Some strategies for the improvement of ZnO properties were discussed such as: doping and co-doping of ZnO films and the addition of polymers, graphene, or other oxide materials in the ZnO matrix. Each approach is discussed in connection with the intended application. In order to prepare sensing (gas or biomarker) or transparent conductive materials the incorporation of one or more dopants is necessary, to induce p- or n-type conductivity, while the piezoelectricity is improved by growing 2D structures or polymer coatings.

The future major challenges regarding the development of sensitive materials consist of a better understanding of the sensing mechanism (gas sensor: adsorption reactions; biosensor: host–guest interactions), improving the sensitivity, selectivity, and stability of the samples as well as reducing the operating temperature to room temperature. An expanded analysis concerning the increase of gas performance of ZnO-based materials can be conducted through (a) control of morphology (optimization of synthesis parameters), (b) defects generation (finding the suitable dopant concentration), (c) investigation of photophysical (photons generation under excitation) and photochemical properties (generation of electrons after excitation) and (d) development of new composite materials (creating of surface defects which can lead to a better adsorption by electron transfer).

In the case of biological applications, additional studies are needed to elucidate the interaction mechanism between the sample and analyzed species and the study of biocompatibility of ZnO composite materials (implants or stents). From a biological point of view, an important challenge is the immobilization of the biomolecules on the surface of the sensor, while the extinction of the fluorescence would be a major problem for the photoluminescent sensors. The piezoelectricity of ZnO-based systems remains a promising topic for medical applications. An interesting approach to achieving piezoelectric properties involves the growth of nanostructures with preferential orientations and good uniformity coated with polymer layers, the number of layers having an important influence on the piezo–response of the final materials. As mentioned above, there are still many aspects that need to be to be further investigated in order to improve the performance of the studied materials.

In conclusion, due to its versatility, ZnO has gained a great interest in the scientific community since its discovery and will be studied in the future for a wide range of applications.

## Figures and Tables

**Table 1:** ZnO in composite layers deposited by chemical methods for sensor applications.

No.	Hetero-Structures	Method	Year	Sensor Application	Main Results	Ref.
1	ZnO-NiO	HT	2019	H_2_S	The performance of the gas sensor toward H_2_S was significantly improved after the formation of NiO/ZnO heterostructures.	[144]
2		HT	2020	VOC	Selective VOCs sensors based on NiO/ZnO p–n heterojunction diode for 2-propanol, toluene, and formaldehyde vapors detection can be attained by controlling the applied voltage. An advantage of this diode is the ability to modify the forward bias voltage, tailoring the number of carriers implied in the sensing process. A higher forward voltage leads to the increase of the O^−^ adsorbates that exist on the ZnO surface.	[145]
3		HT	2020	NH_3_	The improvement of gas sensing properties could be assigned to the hierarchical structure which leads to a better adsorption of gas molecules and also the formation of n-ZnO/p-NiO heterojunction.	[146]
4		HT	2022	H_2_CO	The detection of formaldehyde at low temperatures was improved by the formation of ZnO/NiO heterostructures with high porosity which promotes the adsorption of gas molecules on the surface.	[147]
5	CdO-ZnO	HT	2021	H_2_CO	The gas sensing measurements highlighted an improved response of CdO-ZnO nanorices structures towards formaldehyde gas sensing, compared to the ZnO nanoflowers.	[148]
6	ZnO-CuO	SG	2018	H_2_CO	The gas sensing properties of the ZnO sensor can be enhanced through CuO addition to creating a CuO/ZnO heterojunction. The experimental results proved that the CuO/ZnO-based sensor exhibits exceptional selectivity and sensitivity for room temperature formaldehyde detection.	[149]
7	ZnO-graphene	SG	2018	NO_2_	G-ZnO composite thin films act as selective sensors for NO_2_ detection at low temperature, the superior capabilities being due to the concomitant adsorption of NO_2_ gas and molecular oxygen on the graphene and ZnO surfaces.	[150]
8		SG	2021	NO_2_	The hybrid materials based on ZnO/graphene heterostructures improve gas detection sensitivity at low temperatures due to the combination between the specific properties of ZnO and graphene.	[151]
9		Reflux method	2018	CO	The rGO–ZnO composites enhance the sensor performance, in terms of reducing the working temperatures for CO gas detection.	[152]
10	ZnO-SnO_2_	SG	2016	CO	The ZnO–SnO_2_ composite materials with different content of SnO_2_ selectively detect the CO gas.	[153]
11		HT	2019	C_2_H_5_OH	The SnO_2_/ZnO heterostructures show a higher gas sensing response in contrast with the ZnO nanorods. The formation of SnO_2_/ZnO heterojunction may be responsible for the improved performance of the sensors.	[154]
12		Spray pyrolysis	2019		The Zn:Sn molar ratio has an important role in the morphology of the nanostructures, the best gas sensing results being obtained in the case of a higher content of ZnO nanorods. Thus, a better sensitivity was found in the films with higher amounts of ZnO, due to their higher crystallinity.	[155]
13	ZnO-Cr_2_O_3_	Two-step chemical route	2018	H_2_CO	The gas sensing measurement showed that the Cr_2_O_3_-ZnO heterostructures exhibit excellent gas sensing properties for formaldehyde, which can be assigned to the formation/presence of hierarchical structures.	[156]

**Table 2:** Effect of Zn doping on different oxides prepared by SG.

Year	Dopant Ions	Doped Oxide	Doping Effect	Ref.
2022	Co^2+^, Cu^2+^, Zn^2+^	NiO	Changes in the NiO film color	[170]
2022	Zn^2+^	MgO	Biosensors-detection of glucose level	[171]
2022	Zn^2+^	ITO	Improved sensor response to CO_2_ and TCO characteristics for solar cell	[172]
2022	Zn^2+^	SrTiO_3_	Good effect on the dielectric response	[173]
2022	Zn^2+^	ITO	Optimized electrical conductivity and carrier density	[174]
2021	Zn^2+^	NiO	Refractive index increase with the Zn concentration (1–5%)	[175]
2018	Zn^2+^	BiFeO_3_	Significant decrease of the leakage current of BiFeO_3_ film at low electric fields.	[176]
2018	Zn^2+^	CuO	Increasing band gap with Zn concentration	[177]
2017	Cu^2+^, Zn^2+^, Mn^2+^	BiFeO_3_	Considerably lower leakage currents in doped films compared with pure BFO film	[178]

**Table 3:** The ionic character of some elements used for doping ZnO.

Ionic Character			
Acceptor	Donor		Acceptor-Donor
Li [197,199,200,201,202,203]	F [204,209,214]	Te [206,207]	Li-Ni [199]
Na [192,197,204,205]	Cl [214]	Ga [214,215]	Ga-N [213]
K [197]	Al [216,217]	Ni [199,208,217]	In-N [203]
N [193,194,212]	In [196]	Mn [218]	Al-N [210]
P [194]	S [206]		F-Ag [195]
Sn [215]	Se [206]		

**Table 4:** An overview of the SG-ZnO applications in the last ten years.

Year	Title	Application	Review Content	Ref.
2023	Controlled Growth of Semiconducting ZnO Nanorods for Piezoelectric Energy Harvesting-Based Nanogenerators	Piezoelectric Nanogenerator; Energy harvesting	ZnO nanorods; Piezoelectric properties; Piezoelectric devices;	[219]
2023	Recent Advances in Integrating 1D Nanomaterials into Chemiresistive Gas Sensor Devices	Gas sensors	1D Nanomaterials; Electrical properties; Gas sensing	[220]
2022	92 years of zinc oxide: has been studied by the scientific community since the 1930s- An overview	Rubber industry; Biosensors; Textile industry; Agriculture (nano-fertilizers)	Vulcanization properties; Biological properties; UV blocking property; photo-catalytic self-cleaning; Electrical conductivity; Photoluminescence (PL) properties; Anti-fungal properties	[1]
2022	A review of flexible lead-free piezoelectric energy harvester	Piezoelectric Nanogenerator; Energy harvesting; Flexible Nanogenerator	ZnO NWs; Electrical properties; Piezoelectric behavior	[52]
2022	Morphological evolution-driven semiconducting nanostructures for emerging solar, biological, and nanogenerator applications	Solar cells; Nanogenerator; Biological applications	ZnO nanostructures; Antimicrobial properties; Antilarvicidal activity; Anticancer activity; Piezoelectric properties	[21]
2022	ZnO Transducers for Photoluminescence-Based Biosensors	Biosensors	PL Properties	[33]
2022	A Review of the Impact of Zinc Oxide Nanostructure Morphology on Perovskite Solar Cell Performance	Solar Cell	Zinc Oxide Nanostructure; Electron mobility	[22]
2022	Immobilization of zinc oxide-based photocatalysts for organic pollutant degradation: A review	Photocatalysis	Photocatalytic activity	[221]
2021	Economic Friendly ZnO-Based UV Sensors Using Hydrothermal Growth: A Review	UV sensors	Piezo-phototronics and piezotronics; conductivity; photoresitivity	[7]
2021	Review of ZnO-based nanomaterials in gas sensors	Sensors	ZnO nanomaterials; ZnO nanocomposite; Gas sensing properties; Electronic properties	[83]
2020	Photoluminescence of ZnO Nanowires: A Review	Photoluminescence applications	ZnO Nws; Optoelectronic properties; PL properties	[24]
2020	A review on ZnO: Fundamental properties and applications	Field effect transistors (FET); Gas sensing; LED devices; Environmental applications	ZnO; Optical, magnetic, and PL properties	[222]
2020	Advances in doped ZnO nanostructures for gas sensor	Gas sensors	ZnO nanostructures; Metal doping; Hetero atomic doping	[223]
2019	ZnO as a Functional Material	Biomarkers; Gas sensors	ZnO p-type; PL	[4]
2019	Enhanced sensing performance of ZnO nanostructures-based gas sensors	Sensors; Gas sensors	ZnO nanostructures; Nanocomposites; Gas sensing properties; Metal doping; UV activation; heterojunction	[224]
2018	Synthesis, properties, and applications of ZnO nanomaterials with oxygen vacancies: A review	Photocatalyst; Photoelectrochemical water oxidation; Antibacterial agents; Gas sensors; Supercapacitors; Electronic devices	ZnO nanomaterials; PL; Electrical properties; Ferromagnetism; Antibacterial activity; Gas sensing properties	[225]
2018	Fabrications and Applications of ZnO Nanomaterials in Flexible Functional Devices-A Review	Solar cell; Supercapacitors; Flexible piezoelectric NGs; UV photodetectors (PDs); Photodiodes; Flexible and porous 3-D ceramics; Functional surface coating; Biosensors; Gas sensors	ZnO nanomaterials; Thin films; Optical and electrical properties	[226]
2017	ZnO Nanowire Application in Chemoresistive Sensing: A Review	Gas sensors; Biosensors	ZnO NWs; ZnO Nanowire Sensors; Sensing, photoresponse, and semiconductor properties	[227]
2017	Zinc oxide nanostructure-based dye-sensitized solar cells	DSSCs	ZnO nanomaterials; Photosensitizer dyes; Photoconversion efficiency	[228]
2016	Optical biosensors based on ZnO nanostructures: advantages and perspectives. A review	Optical biosensors	ZnO nanostructures; Functionalization of ZnO surface	[229]
2015	ZnO nanostructured thin films: Depositions, properties, and applications—A review	Gas Sensors; SAW Devices Thin Film Transistors (TFT); LED; Solar Cells	ZnO thin films; Optical and electrical properties	[230]
2014	Zinc Oxide Nanomaterials for Biomedical Fluorescence Detection	Biomedical	Optical and electronic properties ZnO NR	[231]
2013	p-Type ZnO materials: Theory, growth, properties, and devices	LED; Photodetector; Field-effect transistor (FET); Sensors; Piezoelectric NG	Homo- and heterojunctions p-doping of ZnO films; Emission properties	[232]

## Data Availability

The data presented in this study are available on request from the corresponding author.

## Abbreviations

List of acronyms (in alphabetical order)	
AZO	Al-doped ZnO
BF	Bright field
CBD	Chemical Bath Deposition
CNFs	Ceramic nanofibers
CNT	Carbon nanotubes
CP	Conjugated polymer
CVD	Chemical Vapor Deposition
DMS	Dilute Magnetic Semiconductors
DSSCs	Dye-sensitized solar cells
EC	Ethylcellulose
ETL	Efficient electron transport layer
FET	Field effect transistors
HT	Hydrothermal synthesis
HPC	Hydroxypropyl cellulose
ITO	Indium Tin Oxide
LED	Light emitting diodes
NLO	Nonlinear optical properties
NG	Nanogenerator
NPs	Nanoparticles
NR	Nanorods
NWs	Nanowires
OFET	Organic field-effect transistor
PBTA	Poly(butylene adipate-co-terephthalate)
PDMS	Poly(dimethylsiloxane)
PDOS	Partial density of states
PEBA	Polyether block amide
PEI	Polyethyleneimine
PL	Photoluminescence
PLA	Polylactic acid
PP	Polypropylene
PVA	Poly(vinyl alcohol)
POPs	Persistent organic pollutants
RE	Rare earth
ROS	Reactive oxygen species
rGO	Reduced graphene
RhB	Rhodamine-B
SEM	Scanning Electron Microscopy
SG	Sol-Gel
SILAR	Successive Ionic Layer Adsorption and Reaction
SL	Seed Layer
TCO	Transparent Conductive Oxide
TEM	Transmission electron microscopy
TFT	Thin Film Transistors
UV	Ultraviolet
VOC	Volatile organic compound
